# Divergent Effect of Cigarette Smoke on Innate Immunity in Inflammatory Bowel Disease: A Nicotine-Infection Interaction

**DOI:** 10.3390/ijms21165801

**Published:** 2020-08-13

**Authors:** Dania AlQasrawi, Ahmad Qasem, Saleh A. Naser

**Affiliations:** Division of Molecular Microbiology, Burnett School of Biomedical Sciences, College of Medicine, University of Central Florida, Orlando, FL 32816, USA; daniaqasrawi@Knights.ucf.edu (D.A.); Ahmadqasem@knights.ucf.edu (A.Q.)

**Keywords:** nicotine, Crohn’s disease, ulcerative colitis, macrophage, MAP, miR-124, α7nAChR

## Abstract

Cigarette smoke (CS) has adverse effects in patients with Crohn’s disease (CD), an inflammatory bowel disease (IBD) that has been associated with microbial infection, immuno-dysregulation, and mucosal dysfunction. However, CS seems to provide relief and protection to patients with another IBD known as ulcerative colitis (UC). These two subsets are featured as M1- and M2-mediated responses, respectively. Nicotine is the most active, addictive, and studied ingredient in CS. The mechanism of how nicotine and/or other CS ingredients induce pro-inflammatory or anti-inflammatory phenotypes in IBD patients remains under investigation. Our most recent in vitro nicotine study provided significant insights toward understanding the contradictory effects of nicotine on IBD patients, and it elucidated the mechanistic role of α7nAChR in modulation of macrophages in tobacco smokers. Shifting the beneficial effect of nicotine to a harmful outcome in CD patients was linked to a nicotine-microbe interaction that supports a microbial etiology in CD pathogenesis. Among the most debated pathogens in CD etiology is *Mycobacterium avium* subspecies *paratuberculosis* (MAP). Other studies associated nicotine with upregulation of miR-124 expression in macrophages, which led to anti-inflammatory response. This review discusses published work on the role of nicotine in modulation of the innate immune response and subsequent signaling in macrophages in IBD subsets.

## 1. Introduction

Inflammatory bowel disease (IBD) is a chronic condition that involves severe inflammation in the lining of the digestive tract [1]. IBD, which includes Crohn’s disease (CD) and ulcerative colitis (UC), occurs more commonly in developed countries with an average incidence of 10 per 100,000 persons [1]. General IBD symptoms include weight loss, bloody diarrhea, anemia, fever, rectal bleeding, severe ulceration, and loss of appetite, which ultimately lead to poor quality of life [2,3]. The pathogenesis of IBD is still not fully understood. However, there is compelling evidence that supports the association of IBD in genetically susceptible persons with environmental triggers including infection [4]. There are at least 200 genetic loci that have been identified as targets and may increase susceptibility to IBD [4]. For instance, variants of human leukocyte antigen (HLA) haplotypes as HLA class II allele DRBI*0103 have been strongly associated with UC, whereas nucleotide-binding oligomerization domain-containing protein 2 (NOD2) polymorphisms have been linked to CD [5]. NOD2 is the primary receptor responsible for intracellular bacterial recognition and clearance in tissue [5]. Other factors have been specifically associated with CD pathogenesis, and not UC, such as environmental triggers including diet, UV light exposure, vitamin D deficiency, infection, and tobacco smoking. Infection is well established as a key complication in CD and not in UC pathogenesis [6]. Opportunistic enteric infections and zoonotic-linked pathogens have been reported exclusively in CD patients [4,7,8]. Among the most investigated pathogens in CD etiology is *Mycobacterium avium* subspecies *paratuberculosis* (MAP) [7,8]. This fastidious acid-fast bacillus is responsible for CD-like disease in ruminants with Johne’s disease [9]. On the other hand, tobacco smoking has been reported to have contradictory effects on IBD patients [10]. It alleviates the symptoms and provides protective effect in smokers with UC. The opposite is true in CD smokers; hence, it causes detrimental effect and exacerbates the symptoms [10,11]. Such contradictory effects among IBD patients was initially reported in a 1982 study by Harries et al., who observed protective effects of tobacco smoking in UC patients, while smoking was harmful in patients with CD [10]. This observation has been confirmed and became well accepted by clinicians. The latter now recommend nicotine replacement therapy as a treatment option for some UC patients [10,11]. Smoking tobacco not just always exacerbates symptoms in CD patients, it also causes higher risk of relapse than non-smokers with CD [10,11].

To understand this dogma in IBD subsets, our research team has been investigating the effects of cigarette smoke and nicotine on the cellular and molecular changes in CD- and UC-like macrophages. AlQasrawi et al. reported recently that they were able to mimic macrophages similar to those in CD and UC patients with history of prior or active cigarette smoking and studied cellular response in the presence and absence of active bacterial infection [12]. Among the findings, they elucidated how nicotine in UC active smokers activates the cholinergic anti-inflammatory pathway through α7-nicotinic acetylcholine receptor (α7nAChRs). This caused shift toward M2-macrophage polarization, which resulted in upregulation of anti-inflammatory cytokines Interlukin-10 (IL-10) and downregulation of pro-inflammatory cytokines (IL-6 and tumor necrosis factor alpha (TNF-α)). They reported that decrease in caspase-3 activity was a key factor in macrophage modulation toward anti-inflammatory phenotypic response [12]. On the other hand, the effect of nicotine in macrophages infected with MAP, *M. tuberculosis*, or *Klebsiella pneumoniae* was contrary to that observed in UC-like macrophages [12]. These findings confirm the school of thought that bacterial infection plays a key role in CD pathogenesis. This study will focus more on interplay between infection and smoking and their effects on IBD patients.

## 2. Cigarette Smoke Is Detrimental in Crohn’s Disease

Many studies strongly support smoking as an independent risk factor of CD, and it may even affect the severity of the disease (Table 1). CD patients who smoke or start smoking after diagnosis have higher numbers of relapses requiring greater immunosuppression that can lead to hospitalization, in comparison to patients who quit smoking or never smoked [11,13]. This was reported first in 1984 by Holdstock et al. by analyzing 172 variables [14]. They reported that active CD smokers were more likely to be hospitalized due to a higher number of relapses and increased disease severity [14]. Another study reported the association of smoking with increased risk of relapsing in 152 CD patients (smokers vs. non-smokers), which suggested a twofold increased relapse rate among smokers as compared to non-smokers [15]. Other researchers investigated the effect of dose and duration of smoking in CD patients [16,17]. In a thirteen-year study of almost 3000 CD patients, the harmful effect of smoking on CD patients was dose-dependent, causing heavy smokers to be at higher risk of needing immunosuppressants [16]. In this study, they divided CD patients into three groups: non-smokers, light smokers (1–10 cigarettes per day), and heavy smokers (>10 cigarettes per day), then they determined the annual disease activity and immunosuppressant requirement for each group [16]. Lindberg et al. investigated 231 CD patients and reported that heavy smokers (>10 cigarettes per day) had an increased risk for surgery as well as higher risk of further surgeries 10 years after diagnosis compared to non-smokers [17]. Limited studies reported contradictory observations where active CD smokers showed no negative effects as a result of CS [18].

### Factors Affecting the Severity of Symptoms in CD Smokers

Genetics and/or ethnicity factors have been reported in the literature to influence the degree of how smoking impacts CD severity. Aldhous and Satsangi reported that there was no association between smoking and CD susceptibility among Jewish populations [19]. Another study reported a higher percentage of smokers with CD in French Canadian populations compared to other Caucasian populations [19]. Most recently, researchers adapted different criteria for better CD stratification, such as Vienna and Montreal classifications, which depend on disease location and behavior [19]. Using these criteria, some suggested that smoking can progress CD stage toward fistulas or strictures formation while the location of the disease remains stable [20]. Another study reported that smoking has a strong association with the development of penetrating profile in non-colonic CD [21]. On the other hand, several studies reported a lack of association between smoking and CD location and behavior [22]. Overall, all studies that found a direct link between smoking and CD concluded that smoking is more related to non-colonic and severe CD cases, whereas non-smoking or light smoking may be associated with colonic and benign CD.

## 3. Cigarette Smoke Is a Protective Factor in Ulcerative Colitis

Paradoxically, smoking has been a favorable and protective environmental association in patients with UC [10]. This protective association between smoking and UC was first noted in 1976 by Samuelsson et al., who attributed this observation to interactions with medication [23]. Five years later, Harries et al. confirmed the protective effect of smoking in UC patients by analyzing data from 230 UC patients and 192 CD patients. They concluded that only 8% of UC patients were current smokers compared to 42% of CD patients [24]. They reported that 44% of UC patients and 27% of CD patients were ex-smokers [24]. A British study in 1998 involving 51 UC patients established that smoking has a positive effect on UC [25]. Further supporting evidence of the beneficial effects of smoking on UC were revealed by a subsequent study that concluded that resuming smoking in low doses can be used as a medication instead of steroids in ex-smokers with refractory UC [26]. A recent Hungarian study of 1420 IBD patients, including 914 UC patients and 506 CD patients, found that the effect of smoking on IBD is linked to gender and age, and it is most prominent in young adults [27]. They also showed that the prevalence of smoking is 14.9% in UC patients and 47% in CD patients [27]. This study was in line with a meta-analysis by Mahid et al., who confirmed the protective role of smoking on UC, with a 0.85 odd ratio (OR) of current smokers, as compared to lifetime non-smokers [28]. Further studies investigating the relationship between smoking and UC observed that smoking tends to be dose-dependent in UC patients [29,30]. A study examining 499 UC patients found that relatively heavy smokers with UC have healthier colons and less histological evidence of inflammation in colonoscopy examinations than light smokers [29]. Prior to that, a Japanese study also revealed a significant dose-response relation between smoking and UC among Japanese people [30]. Subsequent studies have confirmed the observation that current smokers with UC have fewer relapses, reduced need for immunosuppressant therapy, and fewer hospitalizations, compared to ex-smokers and non-smokers with UC [23,24,25,26,27,28,29,30] (Table 2).

### Nicotine Replacment

Interestingly, different nicotine replacement forms have been recently recommended by clinicians to help UC patients. This includes transdermal patches, chewing gum, and nicotine-based enemas [31]. In a study where 72 active UC patients were treated with either transdermal nicotine patches or placebo patches for six weeks, they found that transdermal nicotine replacement was significantly more effective than the placebo at reducing the severity of UC. They also reported serious side effects such as headache, nausea, sleep disturbance, and acute pancreatitis [32]. However, using nicotine enema or oral capsule formulations significantly decreased these side effects [33]. Another study claimed that chewing nicotine gum exerted the same positive influence on UC severity as observed in current smokers [34].

## 4. The Contradictory Effect of CS on IBD Subsets Is Due to Nicotine-Multifactorial Interaction

### 4.1. Nicotine and the Other Factors

Tobacco leaf extracts contain roughly 4500 components, and around 150 of those have been labeled as harmful to human health, such as dioxins, which are known to be both carcinogenic and an immunomodulator [10]. Heavy metals such as cadmium and arsenic are also considered to be carcinogenic in addition to their role in the development of gastric ulcers [35]. Despite all the carcinogenic or toxic effects of these components in human health, nicotine remains the most active agent in tobacco that is thought to have the greatest effect. Nicotine is an alkaloid found naturally in the roots and leaves of the nightshade family of plants, such as *Nicotiana tabacum*. Although nicotine itself is not toxic, it is considered a highly addictive component of tobacco [12]. This may explain why most recent studies are mainly focused on establishing the role of nicotine in IBD pathogenesis.

**Figure 1:**
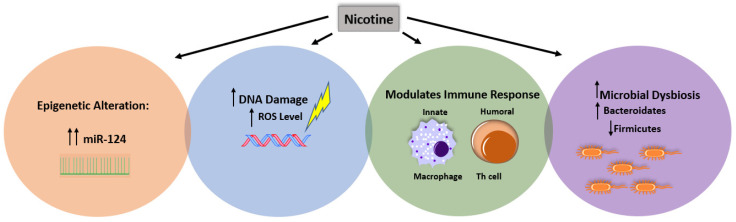
Effect of multi-factorial interaction and nicotine on IBD pathogenesis. Illustration of how nicotine may regulate the pathogenesis of IBD by stimulating DNA damage in intestinal cells, inducing microbial dysbiosis and increasing susceptibility to infection, increasing the probability of epigenetics, and modulating intestinal immune response.

Despite the extensive studies about the involvement of nicotine in IBD pathogenesis, the mechanisms by which nicotine modulates immune response in CD and UC require more elucidation. We propose that these mechanisms require a multi-factorial interaction between nicotine and the immune system, dysbiosis leading to infection, or are epigenetic, as illustrated in Figure 1.

### 4.2. Nicotine and Immune Response

#### 4.2.1. Cytokines

Nicotine is known to have a suppressive effect on both innate and humoral immune systems [12]. Many studies showed that nicotine inhibits TNF-α release from mononuclear cells isolated from healthy volunteers, while it stimulates the secretion of IL-10, which is known as an anti-inflammatory cytokine [12]. The inflammatory process in CD has been looked at predominantly as a humoral response by activation of Th1/Th17 cells, which in turn converts the inflammatory response to be more harmful [3]. Moreover, smoking leads to an elevation of pro-inflammatory cytokines including TNF-α, interferon gamma (IFN-γ), IL-23, IL-6, and IL-1β, both in the gut and peripheral blood of CD patients [3]. Consequently, this exacerbates inflammation and triggers CD symptoms [3]. Meanwhile, the effect of nicotine on innate immunity (monocyte/macrophage) in CD remains to be elucidated.

#### 4.2.2. α7nAChR in Immune Cells

It is well-known that nicotine activates cells through α7nAChR, which is responsible for the activation of cholinergic anti-inflammatory pathway [12]. The presence of α7nAChR on the cell surface of monocytes and macrophages adds to the role of nicotine in innate immune response [12,36]. Infection triggers acetylcholine (Ach) release by the vagal nerve, which binds with α7-nAChR on the surface of macrophages and subsequently interferes with the production of pro-inflammatory cytokines [37]. Surprisingly, nicotine has the same effect as that of vagal nerve stimulation, leading to anti-inflammatory response by shifting the macrophage polarization toward M2, weakening the production of pro-inflammatory cytokines and increasing the secretion of anti-inflammatory cytokines as well [12,37]. Moreover, activation of α7nAChR in CD4+ CD25+ regulatory T-cells is responsible for an immunosuppressive effect by producing IL-2 and downregulating nuclear factor kappa-light-chain-enhancer of activated B cells (NF-ҝB) [37]. To confirm this, other studies claimed that the presence of α7nAChR in endothelial cells may help in reduction of chemokines and adhesion molecules during inflammation [38]. These findings may explain again the cholinergic anti-inflammatory protective effects of nicotine among UC patients. However, limited studies have looked at how the innate immune response interacts with the overall inflammatory process in IBD and the role of nicotine in macrophage recruitment, proliferation, and differentiation in CD vs. UC.

#### 4.2.3. Microbial Dysbiosis

The gut microbial diversity and load have been studied in IBD. Dysbiosis occurs in IBD as defined by decrease in gut microbial diversity with shifting the balance between beneficial and pathogenic bacteria, which may lead to aberrant immune response in genetically susceptible individuals [39]. In CD patients, enteric dysbiosis is common, which is represented by significant reduction in commensal phyla such as Firmicutes and Actinobacteria, with a higher presence of proteobacteria phyla [40]. Many studies have looked to the role of microbial dysbiosis in initiation of pathogenic infection such as *Clostridium difficile* in CD [41]. However, further investigation is needed to elucidate the effect of disturbance of intestinal microbial equilibrium in inducing MAP infection in CD. Among UC patients, gut dysbiosis has been also reported, but the bacterial diversity was not disrupted as much as in CD [40]. Many recent studies have demonstrated the role of the gut microbiome as a link between smoking and CD [10,42,43]. Significant increases in Bacteroides-Prevotella abundance in smoker CD patients compared to non-smokers have been detected through fluorescent in situ hybridization using 16S rRNA sequencing [42]. On the other hand, smoking cessation resulted in a high abundance of Firmicutes (*Clostridium coccoidos*, *Eubacterium rectale*, and *Clostridium leptum*), Actinobacteria (high guanine and cytosine content bacteria (*Propionibacteriaceae* and *Bifidobacteria*)), and a decrease in Bacteroidates (*Prevotella* spp. and *Bacteriods* spp.) [43]. This observation may prove that nicotine withdrawal plays a major role in the shifting of microbial composition toward the composition of healthy individuals.

#### 4.2.4. Epigenetic Susceptibility

The term “epigenetic” refers to heritable changes of gene expression events, which is caused independently of genetic information from the primary DNA sequence [44]. The main epigenetic mechanisms include DNA methylation, histone modification, as well as microRNA (miRNA) [44]. Recently, many studies proposed that epigenetic mechanisms could improve our understanding of IBD, which may lead to new insights of treatment options [44].

Noncoding microRNAs (miRNAs) pathways such as miRNA-18b (miR-18b), miRNA-140 (miR-140), and miRNA-124 (miR124) explain one of the epigenetic mechanisms by which nicotine regulates gene expression of pro-inflammatory cytokines [45]. miR-124 was claimed to be a critical mediator of inflammation that negatively controls its progression [45]. In many cases, it has been shown that nicotine can specifically upregulate miR-124 levels via α7nAChR in macrophages during sepsis [45]. Subsequent studies demonstrated that nicotine could exert an anti-inflammatory response in DSS colitis (resembling UC) by elevating miR-124 levels [46]. However, elevation of mir-124 in TNBS-induced experimental colitis (resembling CD) was found to worsen the inflammation, while blocking miR-124 could alleviate the symptoms [47].

Nicotine has been shown to be responsible for miR-124 elevation in samples in both UC and CD, while the latest has contradictory effect in the two diseases [48]. Recently, a study by Qin et al. demonstrated that miR-124 might mediate the bivalent effect of nicotine on IBD by shifting the Th1/Th2 balance toward Th1 [48]. Consequently, miR-124 could be a suitable target for the contrasting treatment of IBD, up-regulation in UC and down-regulation in CD. Overall, a broad knowledge about the role of nicotine in epigenetic modifications in IBD patients is still lacking. However, this could offer novel treatment options for IBD patients, considering miRNAs as potential therapeutic targets.

## 5. Differences in Immunological Response Profile between Crohn’s Disease and Ulcerative Colitis

One of the most debated explanations for the contradictory effect of nicotine is the association of CD with microbial infection, which lacks in UC. Among the most discussed pathogens associated with CD is MAP [7,8,49,50,51,52,53,54,55,56,57,58].

**Figure 2:**
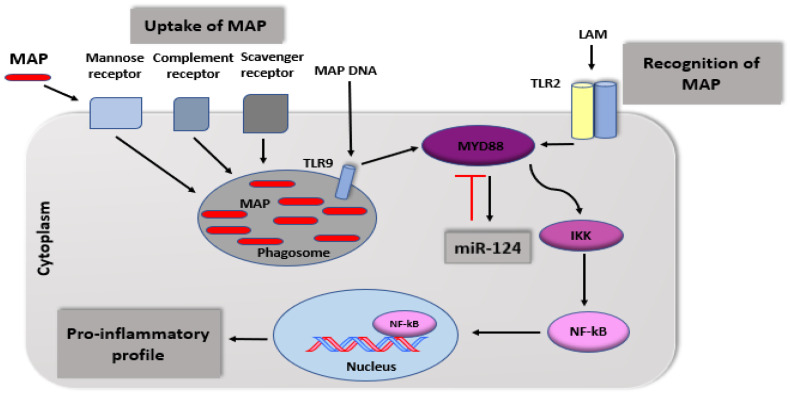
Immune recognition and phagocytosis of MAP in CD macrophages.

MAP is an intracellular pathogen capable of modulating the innate immune response in CD leading to significant production of pro-inflammatory cytokines [7]. It was reported that monocytes and macrophages in CD patients express toll-like receptor (TLR) at higher levels compared with UC patients [59]. TLRs are pattern recognizing receptors in innate immune cells where the cytoplasmic domain is linked to myeloid differentiation primary response-88 (MYD-88). The latter activates transcription factors such as NF- ҝB to produce pro-inflammatory cytokines. Although there are at least 10 types of TLR, TLR2 binds to MAP through the cell wall lipoarabinomannan (LAM), which initiates the activation of the NF-ҝB pathway and production of pro-inflammatory cytokines [60]. Different types of receptors are responsible for MAP uptake, such as scavenger and mannose receptors as well as complement receptors, which are mainly responsible for internalizing opsonized MAP. TLRs are considered the sole receptors for immune response and activation of the pro-inflammatory pathway by recognition of lipoarabinomannan in the surface of macrophages mimicking CD macrophages. Figure 2 shows illustration of how MAP and its cell wall components interact with TLR during phagocytosis and inflammatory response.

The effect of nicotine on IBD patients is clearly demonstrated through changes to gastrointestinal tissue. However, the effect of nicotine and smoking on the respiratory tract is well established. The similarity between intestinal inflammatory response in CD smokers and others with pulmonary inflammation is well studied and reported in the literature. Specifically, the effect of smoking on tuberculosis (TB) and exacerbating of infection has been well elucidated [12,40]. For example, TB smokers are known to have higher levels of alveolar macrophages compared to non-smokers and former TB smokers [61,62]. Bai et al. demonstrated that nicotine impairs anti-MTB defense leading to increase survival and burden of the bacterium in macrophages through α7nAchR by decreasing apoptosis and activation of the NF-ҝB family [63]. We reported similar findings when we studied the effect of nicotine on macrophages infected with related microorganisms, such as MAP [12]. We validated our findings in CD- and UC-like macrophages, and our conclusion should help explain the contradictory effect of smoking on IBD subsets.

**Figure 3:**
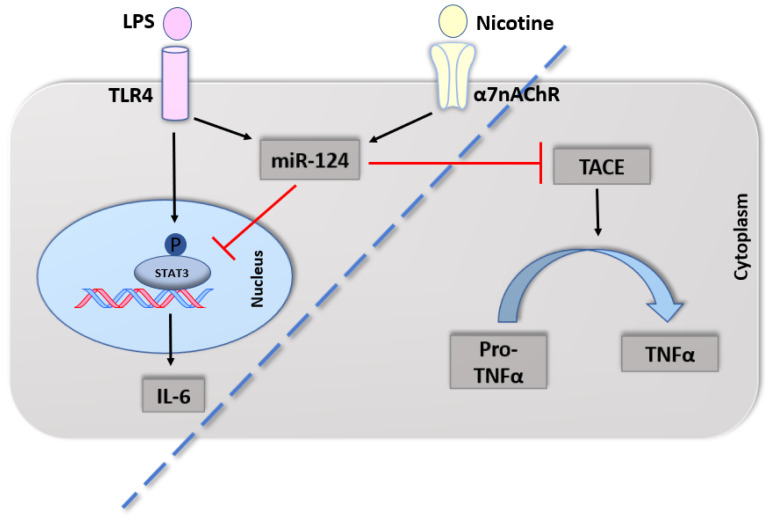
Nicotine activates the anti-inflammatory response in UC macrophages. Nicotine through its receptor, α7nAChR, upregulates miR-124 in LPS-induced macrophages. MiR-124 in turn targets phosphorylated STAT3 and decreases production of IL-6 at the transcriptional level. Meanwhile at the post-transcriptional level, miR-124 blocks TACE, resulting in interruption of TNFα maturation mimicking UC macrophages. Interrupted line to separate cytoplasmic event from nucleic one. STAT3: Signal transducer and activator of transcription 3, TACE: Tumor necrosis factor (TNF)-alpha converting enzyme.

Several mechanisms may explain the protective effect of nicotine on innate immunity in UC. First, nicotine’s effect is mediated by activation of the cholinergic anti-inflammatory pathway via α7nAChR, on macrophages, monocytes, and dendritic cells [40]. This causes a shift in macrophage polarization towards M2 and elevation of IL-10 and decrease pro-inflammatory cytokines levels such as IL-6, IL-12, and TNF-α [38]. Other studies reported that T cells possess α7nAChR, which can be activated by nicotine in Th2 cells and Treg cells, therefore inducing them to produce IL-10 and TGF-β [40]. Second, nicotine was also found to induce production of miR-124, which has an inhibitory effect on pro-inflammatory cytokine production in LPS-induced macrophages [45]. MiR-124 inhibits the translation of TNF-α converting enzyme, therefore preventing the conversion of pro-TNF-α to TNF-α [45] (Figure 3).

## 6. Summary and Outlook

Activation of α7nAChRs downregulates the production of pro-inflammatory cytokines, including TNFα, IL-6, and IL-1β [64]. The downstream signaling to these receptors in macrophages involves NF-kB pathway, whereas in T cells the anti-inflammatory effect requires further investigation, although stimulation of α7nAChRs in T cells showed anti-inflammatory effects [38,65]. Several epidemiological studies showed a clear lower correlation between smokers and the incidence of UC in particular [66]. Nicotine was found as the active ingredient in CS, which is responsible for this immunosuppressive mechanism [67]. On the other hand, CS was associated with higher incidence of numerous inflammatory disorders including rheumatoid arthritis (RA), atherosclerosis, peptic ulcer disease, and CD [66]. This complex effect of nicotine in inflammation is not well understood, but we speculate that the presence of certain pathogens will shift the anti-inflammatory role of nicotine to pro-inflammatory factor.

**Figure 4:**
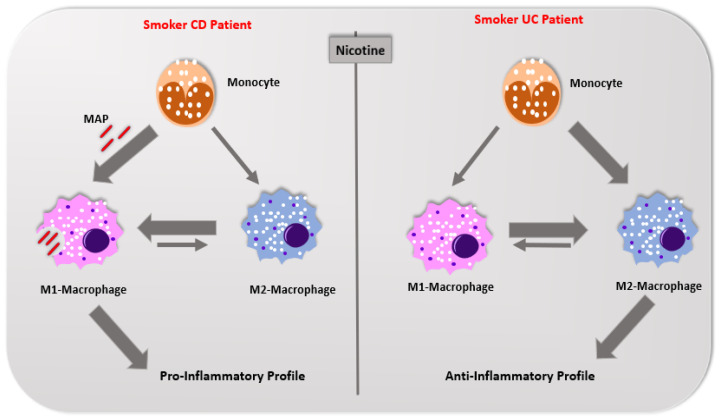
Schematic illustration of the effect of cigarette smoke and nicotine on modulating macrophage polarization in IBD patients.

In conclusion, CS plays a contradictory role in IBD subsets; it is a risk factor in CD development and provides protection in UC. Our most recent in vitro study was the first to provide significant insights toward understanding the cellular events involved in the mysterious divergent effects of CS ingredients on IBD patients [12]. Nicotine exacerbates MAP infection in macrophages in CD patients causing shift toward M1 polarization and excessive production of pro-inflammatory cytokines. The opposite is true in UC; nicotine activates the cholinergic pathway providing anti-inflammatory effect, which provides a treatment option for many of UC patients. Figure 4 illustrates the effect of nicotine on macrophage modulations in IBD smokers. Studying the molecular process and the crosstalk between TLR signaling and miR-124 will further help in understanding why CS is beneficial to UC patients and harmful to those with CD.

## Figures and Tables

**Table 1:** List of clinical studies investigating the effect of tobacco smoking on CD patients.

Study Reference & Published Year	National Origin	Type of Study	Type of Enrollment	Number of Participants	Main Conclusion
Holdstock et al. (1984) [14]	UK	Case-Control Study	Retrospective	172 IBD patients	Smoking leads to CD rather than UC
Somerville et al. (1984) [13]	UK	Case-Control Study	Retrospective	82 variables	CD patients are more likely to smoke
Timmer et al. (1998) [15]	Canada	Cohort	Prospective	152 CD patients	Increased rate of relapses in CD smoker patients
Lindberg et al. (1992) [17]	France	Case-Control Study	Retrospective	231 CD patients	CD heavy smokers have an increased risk of surgery
Seksik et al. (1995–2008) [16]	France	Cohort	Prospective	3000 CD patients	Smoking has a dose-dependent effect on CD patients
Van der Heide et al. (2009) [18]	Netherland	Case-Control Study	Retrospective	820 CD patients	No unfavorable effects of active smoking on CD

**Table 2:** List of clinical studies investigating the effect of smoking on UC patients.

Study Reference	National Origin	Type of Study	Type of Enrollment	Number of Participants	Main Conclusion
Samuelsson et al. (1976) [23]	Sweden	Unknown	Unknown	Unknown	Low rate of smokers among UC patients
Harries et al. (1982) [24]	UK	Cohort	Prospective	230 UC patients	Low rate of smokers among UC patients
Nakarnura et al. (1994) [30]	Japan	Case-Control Study	Retrospective	384 UC patients	The relationship between smoking and UC is dose-dependent
Green et al. (1998) [25]	UK	Cohort	Prospective	51 UC patients	UC is a non-smoker disease
Aldhous et al. (2007) [29]	UK	Case-Control Study	Retrospective	499 IBD patients	UC heavy smokers have healthier colons than UC light smokers
Calabrese et al. (2012) [26]	US	Case-Control Study	Retrospective	15 UC patients	Low doses of smoking can be used as a medication in ex-smoker refractory UC patients
Lakatos et al. (2013) [27]	Hangiri	Cohort	Retrospective	1420 IBD patients	Smoking prevents colectomy in UC smokers
